# Guilingji Protects Against Spermatogenesis Dysfunction From Oxidative Stress via Regulation of MAPK and Apoptotic Signaling Pathways in Immp2l Mutant Mice

**DOI:** 10.3389/fphar.2021.771161

**Published:** 2022-01-13

**Authors:** Zhenqing Wang, Yun Xie, Haicheng Chen, Jiahui Yao, Linyan Lv, Yanqing Li, Chunhua Deng, Min Zhang, Xiangzhou Sun, Guihua Liu

**Affiliations:** ^1^ Department of Urology, The First Affiliated Hospital of Sun Yat-sen University, Guangzhou, China; ^2^ Reproductive Centre, The Sixth Affiliated Hospital, Sun Yat-sen University, Guangzhou, China

**Keywords:** oxidative stress, spermatogenesis, guilingji, Immp2l mutant mice, apoptosis

## Abstract

Male infertility is a major health issue with an estimated prevalence of 4.2% of male infertility worldwide. Oxidative stress (OS) is one of the main causes of male infertility, which is characterized by excessive reactive oxygen species (ROS) or lack of antioxidants. Meanwhile, it is reported that oxidative stress plays an important role in the spermatogenic impairment in Inner mitochondrial membrane peptidase 2-like (Immp2l) mutant mice. In this study, we focused on the potential mechanism of Guilingji in protecting the spermatogenic functions in Immp2l mutant mice. The results revealed that Immp2l mutant mice exhibit impaired spermatogenesis and histology shows seminiferous tubules with reduced spermatogenic cells. After administration of Guilingji [150 mg/kg per day intragastric gavage], however, alleviated spermatogenesis impairment and reversed testis histopathological damage and reduced apoptosis. What’s more, western blotting and the levels of redox classic markers revealed that Guilingji can markedly reduce reactive oxygen species. Moreover, Guilingji treatment led to inhibition of the phosphorylation of mitogen-activated protein kinase (MAPK), regulated apoptosis in the cells. In summary, Guilingji can improve spermatogenesis in Immp2l mutant mice by regulating oxidation-antioxidant balance and MAPK pathway. Our data suggests that Guilingji may be a promising and effective antioxidant candidate for the treatment of male infertility.

## Introduction

Infertility is defined as having regular and unprotected sex with the same partner and not getting pregnant after 1 year (Niederberger et al., 2001). Half of infertility cases are male reproductive dysfunction (Holden et al., 2005), and idiopathic oligasthenospermia is the clinical phenotype (Irvine, 1998). There are many factors affecting sperm quality, including endogenous and exogenous factors. While, many studies have shown that oxidative stress should be considered as a reasonable cause of idiopathic male infertility (Sheweita et al., 2005). Oxidative stress can cause loss of membrane integrity, increased cell permeability, enzyme inactivation, DNA structure damage and cell death, which may be related to decreased sperm count and motility (Atig et al., 2012; Agarwal et al., 2014). At present, the treatment of infertility, especially idiopathic oligasthenospermia, is still mainly drug therapy. Traditional Chinese medicine (TCM) has been attracted more and more attention in recent years for its mild and lasting effects in regulating physiological functions (Duan et al., 2017; Xiao et al., 2019).

Guilingji is a TCM from the Ming Dynasty. According to the 2015 edition of the Chinese Pharmacopoeia, one of the confidential products of national brands of TCM with Red ginseng as the main raw material (Zhao et al., 2017). It has been used for lasting good health and longevity with processing various bioactivities, including anti-aging (Zhao et al., 2020), epilepsy (Liu et al., 1990), testicular dysfunction (Zhao et al., 2019). However, the mechanism of action that produces the above effects is still unclear. We presume that it is related to the balance of oxidative stress during spermatogenesis.

In order to test this hypothesis, we selected Immp2l mutant mice as our animal model. Inner mitochondrial membrane peptidase 2-like (IMMP2L) is a functional domain of mitochondrial intima proteina that plays a crucial role in cleaving space-sorting signal peptide sequences of cytochrome c1 (CYC1) and mitochondrial glycerol phosphate dehydrogenase 2 (GPD2) (Lu et al., 2008; Yang et al., 2016; Du et al., 2020). Therefore, it is an oxidative stress state in the Immp2l mutant mice. Some studies have shown that oxidative stress mediates age-dependent spermatogenesis damage in Immp2l mutant mice (Yang et al., 2016; Du et al., 2020). Thus, we employed the Immp2l mutant mice to investigate the protective effects of Guilingji on oligospermia induced by oxidative stress.

In this study, Immp2l mutant mice were used as the animal model of oligosthenospermia, and Guilingji was selected as the treatment drug by using the unique characteristics of traditional Chinese medicine to explore the influence on these specific antioxidative pathway on oligosthenospermia in mice. This research will provide valuable insights for understanding the pathological changes and treatment mechanisms of spermatogenic dysfunction caused by oxidative stress.

## Materials and Methods

### Medicine and Reagents

The main components of Guilingji (Shanxi Guangyuyuan Medicine Co., Ltd., Lot No. 20151108) are presented in Table 1. Guilingji meets the standard requirements as described in the Chinese Pharmacopoeia (Version 2015) (Zhao et al., 2019). Before the experiment, Guilingji was dissolved in 0.5% sodium carboxymethyl cellulose (CMC-NA) suspension to prepare 30 mg/ml suspension.

**TABLE 1:** The main Chinese medicinal herbs contained in Guilingji.

Chinese name	Latin name	English name	Use part	Properties
Hong Shen	Radix ginseng rubra (*Panax ginseng* C.A.Mey.)	Red ginseng	Root	Warm; mild; bitter
Lu Rong	Cervi cornu pantotrichum	Hairy antler	Non-ossifying young horn of male deer or stag	Warm; sweet; salty
Hai Ma	Hippocampus	Sea-horse	Hippocampus	Warm; sweet; salty
Gou Qi Zi	Fructus lycii (*Lycium barbarum* L.)	Fruit of Chinese wolfberry	Fruit	Mild; sweet
Ding Xiang	Flos caryophylli (*Syzygium aromaticum* (L.) Merr. & L.M.Perry)	Clove	Flower bud	Warm; pungent
Chuan Shan Jia	Manis pentadactyla (*Manis pentadactyla* Linnaeus)	Pangolin	Scale	Minor cold; salty
Niu Xi	Radix achyranthis bidentatae (*Achyranthes bidentata* Blume)	Root of twotooth achyranthes	Root	Mild; sweet; sour; bitter
Suo Yang	Cynomorium songaricum (*Cynomorium cocci-neum* L.)	Songaria cynomorium	Fleshy stem	Warm; sweet
Shu Di Huang	Radix rehmanniae preparata (*Rehmannia glutinosa* (Gaertn.) DC.)	Prepared rehmannia root	Steamed and sundried root	Minor warm; sweet
Bu Gu Zhi	Psoralea corylifolia (*Cullen corylifolium* (L.) Medik.)	Malaytea scurfpea	Fruit	Extreme warm; pungent; bitter
Tu Si Zi	Semen cuseutae (*Cuscuta chinensis* Lam.)	Dodder seed	Seed	Mild; pungent; sweet
Du Zhong	Cortex eucommiae (*Eucommia ulmoides* Oliv.)	Eucommia bark	Bark	Warm; sweet
Rou Cong Rong	Herba cistanches (*Cistanche deserticola* Ma)	Desertliving cistanche	Fleshy stem	Warm; sweet; salty
Gan Cao	Radix glycyrrhizae (*Glycyrrhiza inflata* Batalin)	Root of ural licorice	Root and rhizome	Mild; sweet
Tian Dong	Radix asparagi (*Asparagus cochinchinensis* (Lour.) Merr.)	Cochinchinese asparagus root	Root	Cold; sweet; bitter
Sha Ren	Fructus amomi (*Amomum villosum* Lour.)	Villous amomum fruit	Ripe fruit or seed	Warm; pungent
Fu Zi	Radix aconiti lateralis (*Aconitum carmichaeli* Debeaux)	Common monkshood daughter root	Daughter root	Extreme hot; pungent; sweet
Di Gu Pi	Cortex lycii radicis	Root - bark of Chinese wolfberry	Velamen	Cold; sweet

### Animals and Experimental Design

Immp2l mutant mice have been described previously (Lu et al., 2008). Mice were raised in isolation cages with light/dark cycles of 12 h. The genotype is determined by coat color. Homozygous normal mice became albino mice due to FVB/NJ males (FVB) background. Heterozygous mice were slightly pigmented. Homozygous mutant mice had dark fur color. The animal experiments were carried out in accordance with the Helsinki Declaration and approved by the Laboratory Animal Welfare and Ethics Committee of Sun Yat-sen University.

A total of 40 animals were included in this experiment, including 20 Immp2l^+/+^ mice and 20 Immp2l^-/-^ mice. There were 4 groups in this experiment: Immp2l^+/+^ group; Immp2l^+/+^-Guilingji group; Immp2l^-/-^ group; Immp2l^-/-^-Guilingji group. And there were 10 mice in each group. Immp2l^-/-^ and Immp2l^+/+^ mice were administered intragastrically with Guilingji for 30 days, respectively. The appropriate therapeutic doses of Guilingji is 150 mg/kg per day (Du et al., 2020; Zhao et al., 2020). Mice that received the 0.5% CMC-NA suspension were used as untreated controls. The mice were euthanized by the inhalation of an overdose of isoflurane after treatment. All the mice were weighed each 3 days, that was aimed to reflect the fundamental survival state of the mice.

### Analysis of Sperm Motility and Concentration

After euthanasia of mice, the caudal epididymis of the mice was shredded and suspended in the sperm incubation solution at 37°C for 10–20 min. Then, the sperm suspension was placed on the counting chamber. Sperm count, concentration and motility were measured using a computer assisted sperm analysis system. Then, we place the sperm suspension on the counting chamber, and use computer assisted sperm analysis system to measure sperm count, concentration and motility (SPERM CLASS ANALYZER). a, b, c, d grade respectively represents different motility states of sperm. Sperm motility is quantitatively evaluated by motile sperm (grade a + b)/all sperm*100.

### Testicular Histology

After the mice were euthanized, fresh testicular tissue was taken and cleaned with cold PBS (pH 7.4). Then, fixed in formalin for 48 h and embedded in paraffin after conventional treatment. The wax block was cut into 5 μm thin slices with a microtome, and the slices were stained with hematoxylin and eosin, which were scanned by a light microscope.

### Western Blotting Analysis

Fresh testicular tissue was taken, Radio Immunoprecipitation Assay (RIPA) lysis buffer containing phosphatase inhibitors and protease was added, and total protein was extracted from the tissue after centrifugation. Western blotting analysis was based on previous reports (Yang et al., 2016). Concisely, the denatured proteins were added to the polyacrylamide gel (PAGE) for electrophoresis, and then electrically transferred to polyvinylidene difluoride (PVDF) membranes. This membrane was blocked use non-fat milk at room temperature for 1 h. Next, the membrane was incubated overnight with primary antibody (Table 2) at 4°C. After that, the membrane was rinsed with Tris Buffered Saline with Tween (TBST) and incubated with horseradish peroxidase linked secondary antibody (1:2,000) at room temperature for 1 h. Finally, the membrane was incubated with enhanced chemiluminescence substrate to observe the protein bands. To analysis the protein expression level, ImageJ software was used to relatively quantify protein bands from western blot films. Western bolt was replicated for three parallel experiments.

**TABLE 2:** Primary and secondary antibodies used for Western blotting and Immunofluorescence.

Antibodies (Species, IgG type)	Dilution	Distributor
Primary antibody:		
anti-JNK	1:1,000	Cell signaling technology
anti-p-JNK	1:1,000	Cell signaling technology
anti-ERK1/2	1:1,000	Cell signaling technology
anti-p-ERK1/2	1:1,000	Cell signaling technology
anti-p-38	1:1,000	Cell signaling technology
anti-p-p38	1:1,000	Cell signaling technology
DDX4	1:100	Abcam
Secondary antibody:		
anti-GAPDH	1:10,000	Cell signaling technology
Cy3	1:500	Beyotime

### Immunofluorescence Analysis

For immunofluorescence analysis, the paraffin sections were dewaxed, dehydrated, and incubated overnight at 4°C with primary antibodies. Then, the tissues were incubated with secondary antibodies for 1 h in the dark. Finally, the nuclei were counterstained with DAPI for 10 min. Immunofluorescence signals were visualized and recorded using a laser scanning confocal microscope (TCS SP8, Leica, Germany). The primary and secondary antibodies used in this study are listed in Table 2.

### Measurement of SOD, MDA and GSH Levels in Testicular Tissues

After the mice were euthanized, the tissues were homogenized, then placed in cold sterile phosphate buffered saline (PBS). The homogenate was centrifuged at 2,500 rpm/min at 4°C for 10 min, and the supernatant was extracted for further analysis of oxidative stress level. Assays of Malondialdehyde (MDA), Superoxide dismutase (SOD) and Glutathione (GSH) levels in testicular tissues were performed using commercial kits (Nanjing Jian cheng Bioengineering Institute, China). We further analyzed the test data according to the instructions.

### Transferase dUTP Nick End-Labeling Analysis

The TUNEL assay was executed by using a One‐Step TUNEL Apoptosis Assay Kit. First, paraffin-embedded sections were placed in 65°C drying oven, followed by deparaffinized in xylene, then treated with a range of different concentrations of alcohol [100, 95, 85, 70 and 50% ethanol (v/v)] and rinsed with PBS at pH 7.5. The tissue was then treated with protease K solution and refixed with 4% paraformaldehyde solution. The prepared TUNEL reaction mixture was dropped onto the tissue and incubated at 37°C for 1 h in a dark environment. The nuclei were stained with DAPI. TUNEL positive cells were counted under fluorescence microscope.

### Data Downloading and Functional Enrichment Analysis

Traditional Chinese medicine (TCM) prescriptions can effectively delay the progression of disease through the coordination of various compounds and targets (Li et al., 2021). Therefore, it is necessary not only to identify the potential active ingredients of Guilingji, but also to further explore its therapeutic targets. Gene expression data for TCM herbs/ingredients were downloaded from HERB, which is a high-throughput experiment- and reference-guided database of TCM, traditional with its Chinese name as BenCaoZuJian (http://herb.ac.cn) (Fang et al., 2021). Different molecular mechanisms and involved pathways were explored through Kyoto Encyclopedia of Genes and Genomes (KEGG) and Gene Ontology (GO) analyses. First, we downloaded the gene expression data from HERB’s website (http://herb.ac.cn). Then, using Oncomine (https://www.oncomine.org) for Go and KEGG analysis. Go analysis can reveal the main enrichment terms in Biological Process (BP), Cellular Component (CC) and Molecular Function (MF). KEGG analysis can reveal the major enriched signaling pathways. Pathway with enrichment *p* < 0.01 was considered as significant.

### Statistical Analysis

All experiments were performed at least three times and the data are expressed as the mean ± SEM. When the data were normally distributed, one-way ANOVA followed by Tukey test or two-way ANOVA followed by Bonferroni’s multiple comparison test. If these data were not normal distributed, Kruskal-Wallis test was used. GraphPad Prism 7.0 Software (GraphPad Software Inc., CA, United States) was used for Statistical analysis chart. The differences between groups were considered statistically significant when *p* < 0.05.

## Results

### Guilingji Treatment Improves Sperm Quality and Testicular Index in Immp2l Mutant Mice

**FIGURE 1:**
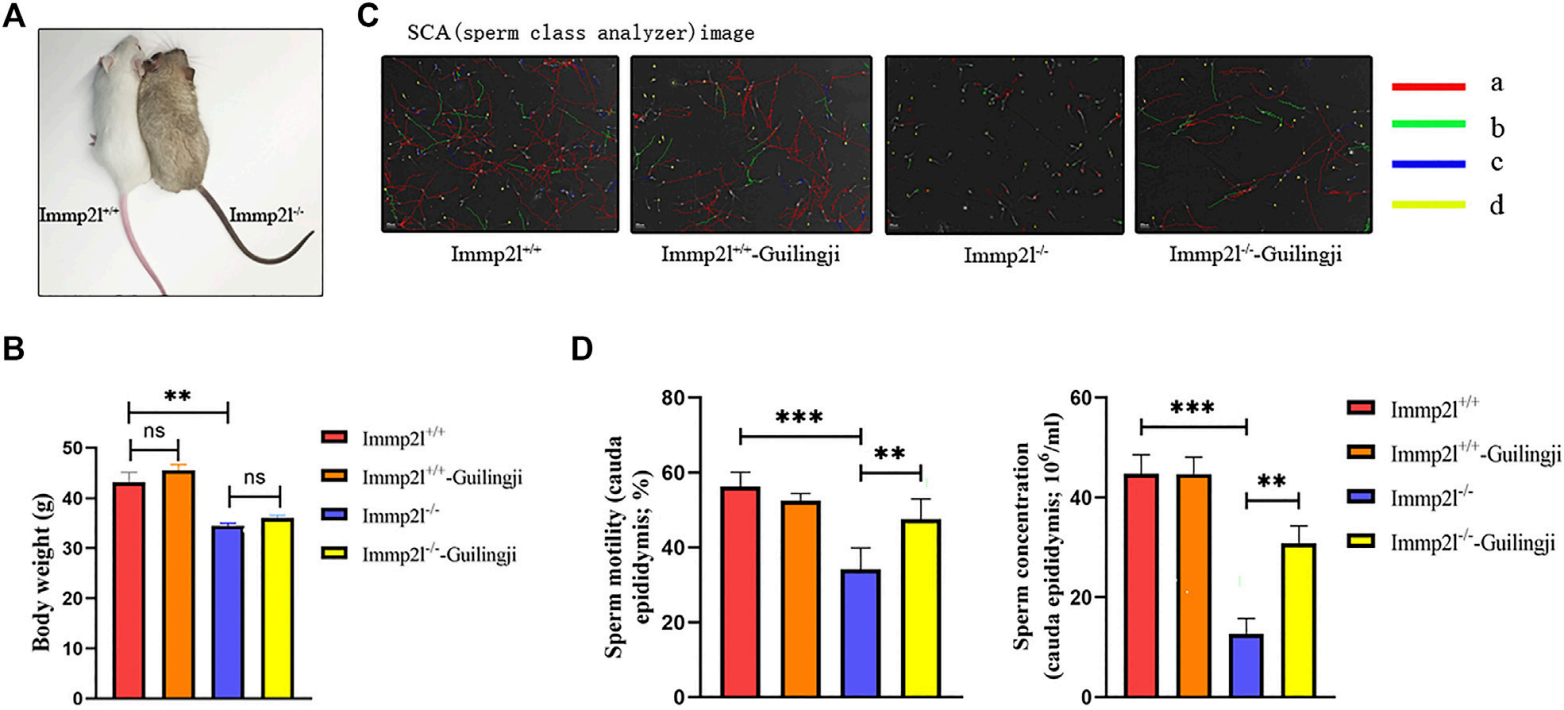
Immp2l mutant mice and sperm quality analysis. **(A)** Homozygous mutant mice were darkly pigmented (right). **(B)** The influence of Guilingji treatment on body weight of Immp2l^−/−^ and Immp2l^+/+^ mice. **(C)** The sperm concentration and motility were measured by a computer-aided sperm analysis system. **(D)** The analysis of sperm concentration and motility. mean ± SEM. ns, Not significant. ** and ***, *p* < 0.01 and *p* < 0.001. *n* = 6.

Mice were monitored for weight loss according to our animal protocol; Immp2l^-/-^ mice weighed less than Immp2l^+/+^ mice (Figure 1B), which is consistent with the results of previous studies (Han et al., 2013). Body weights in Guilingji-treated mice (including Immp2l^+/+^ and Immp2l^-/-^ mice) were not different than in saline-treated mice of the same genotype (Figure 1B).

The detection of sperm motility by Computer-assisted sperm analysis (CASA) was used to evaluate whether Guilingji affected sperm motility and concentration. Results in Figure 1C show the semen picture of Immp2l^-/-^ mice, which revealed that Immp2l^-/-^ mice had lower sperm concentration, motility, and viability. Importantly, the Immp2l^-/-^-Guilingji group displayed a significant increase (*p* < 0.05) in sperm motility percentage and sperm concentration, when compared to the Immp2l^-/-^ group, as presented in Figure 1D.

### Guilingji Treatment Reverses Testis Histopathological Damage in Immp2l Mutant Mice

**FIGURE 2:**
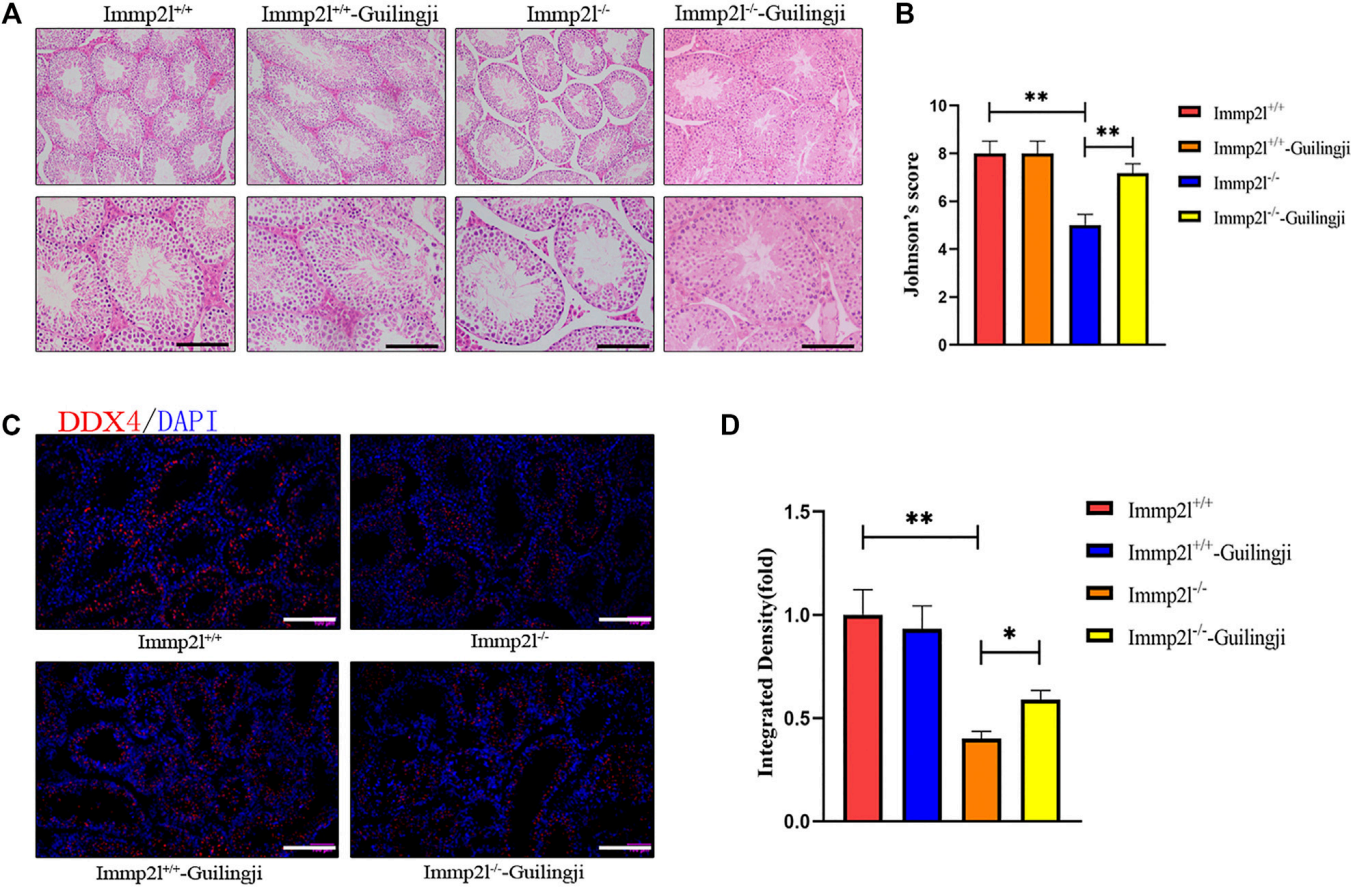
Guilingji treatment repairs testicular damage as assessed by histopathology. **(A)** Transverse sections of testis tissues were stained with hematoxylin and eosin (HE). Scale bar = 20 μm; and **(B)** Spermatogenesis was evaluated by Johnson’s score. **p < 0.01. *n* = 6. **(C)** Immunostaining revealed the expression of DDX4 in testis after Guilingji treatment. The nuclei were counterstained with DAPI (blue). Scale bar = 100 μm.

As shown in Figure 2A, spermatogenesis was normal and spermatogenic epithelial cells were arranged regularly in normal mice. While compared with normal mice, all untreated Immp2l mutant mice showed impaired spermatogenesis, disorganized and vacuolated seminiferous tubules, consistent with oligospermia. In addition, testicular spermatogenesis was evaluated using the Johnsen scores, which is a numerical score from 1 to 10, with 10 indicating complete spermatogenesis. The Immp2l^-/-^-Guilingji group mice showed thicker seminiferous epithelium, and more tubules with the higher Johnsen scores (*p* < 0.05, Figure 2B). It indicated that the testicular structure of the treatment group was less damaged and the function recovered. Furthermore, DDX4 is specific to germ cells, which is necessary for germ cell development. The increased quantity of DDX4-positive cells demonstrated that the spermatogenesis of the experiment animals was improved after the Guilingji administration. As shown in Figure 2C, more DDX4-positive cells were present in the testes of the Immp2l^-/-^-Guilingji group than those in the Immp2l^-/-^ group testes. Summarized data are given in Figure 2D.

### Guilingji Reduces Oxidative Stress in the Testes of Immp2l Mutant Mice

**FIGURE 3:**
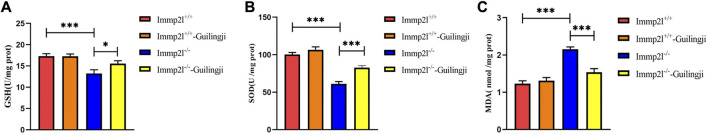
The effects of Guilingji on activities of SOD, GSH and MDA. **(A**–**C)** Activities of SOD, GSH and MDA were detected by kits respectively. The bars showed means ± SEM. *p < 0.05 and ***p < 0.001. *n* = 6.

To assess testicular antioxidant status, we measured the levels of classic markers of redox. As shown in Figure 3, compared with Immp2l^+/+^ mice group, the level of MDA in Immp2l^-/-^ mice group was significantly increased, while glutathione levels and testicular tissue of the superoxide dismutase (SOD) activity were decreased. On the contrary, Guilingji administration can significantly reduce the above effects. The results showed that Guilingji alleviated oxidative stress by balancing oxidation and anti-oxidation.

### Guilingji Protects Testis Tissue Against Apoptosis

**FIGURE 4:**
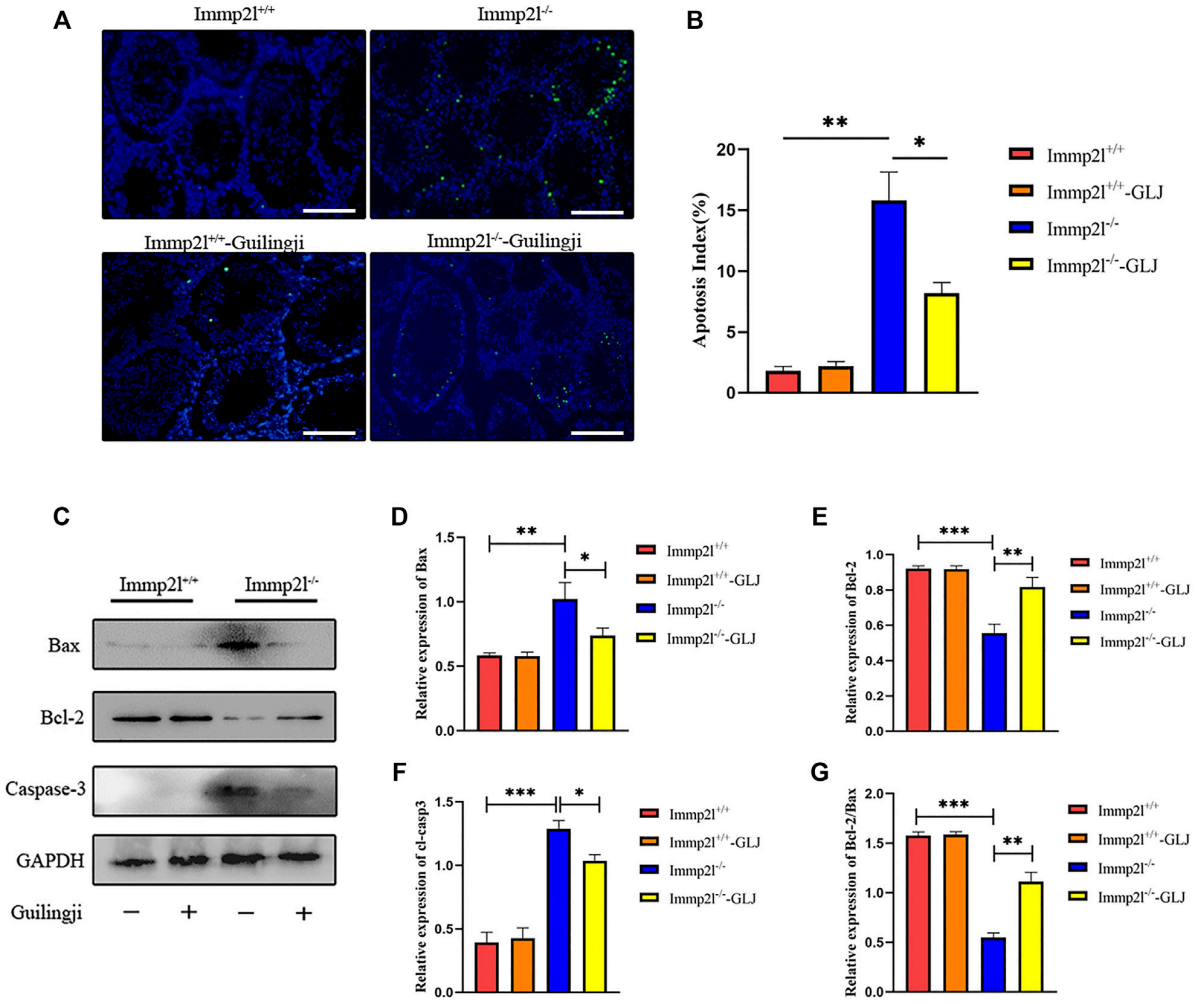
The effects of Guilingji against cell death in testis. **(A)** TUNEL staining. Scale bar = 50 μm. **(B)** The percentage of TUNEL-positive cells within all cells in the slice. The bars showed means ± SEM. *p < 0.05, **p < 0.01. *n* = 5. **(C)** Western blotting was used to analyse proteins extracted from the testis of mice. The relative intensities of **(D)** Bax, **(E)** Bcl-2, **(F)** Caspase-3 and **(G)** the Bcl-2 to Bax ratio. The bars showed means ± SEM of three independent experiments. *p < 0.05, **p < 0.01, ***p < 0.001. *n* = 3.

TdT-mediated dUTP-biotin nick end labeling (TUNEL) is an assay for the detection of apoptosis. As shown in Figure 4A,B, compared with non-mutant mice, the testicular germ cell apoptosis of Immp2l^-/-^ mice was dramatically increased. After Guilingji treatment, the level of apoptosis of germ cells were considerably strangled in Immp2l^-/-^ mice. Meanwhile, Guilingji treatment had no vital effect on the germ cell apoptosis in Immp2l^+/+^ mice. To further confirm this result, we also detected apoptosis-associated proteins active-Casp3, Bcl-2, and Bax expression by western blotting, and found that Guilingji inhibited cleaved Casp3, Bax expression and promote Bcl-2 expression (*p* < 0.05, Figures 4C–F).

### Functional Enrichment Analysis of Ginsenoside Re, the Main Ingredient of Guilingji

**FIGURE 5:**
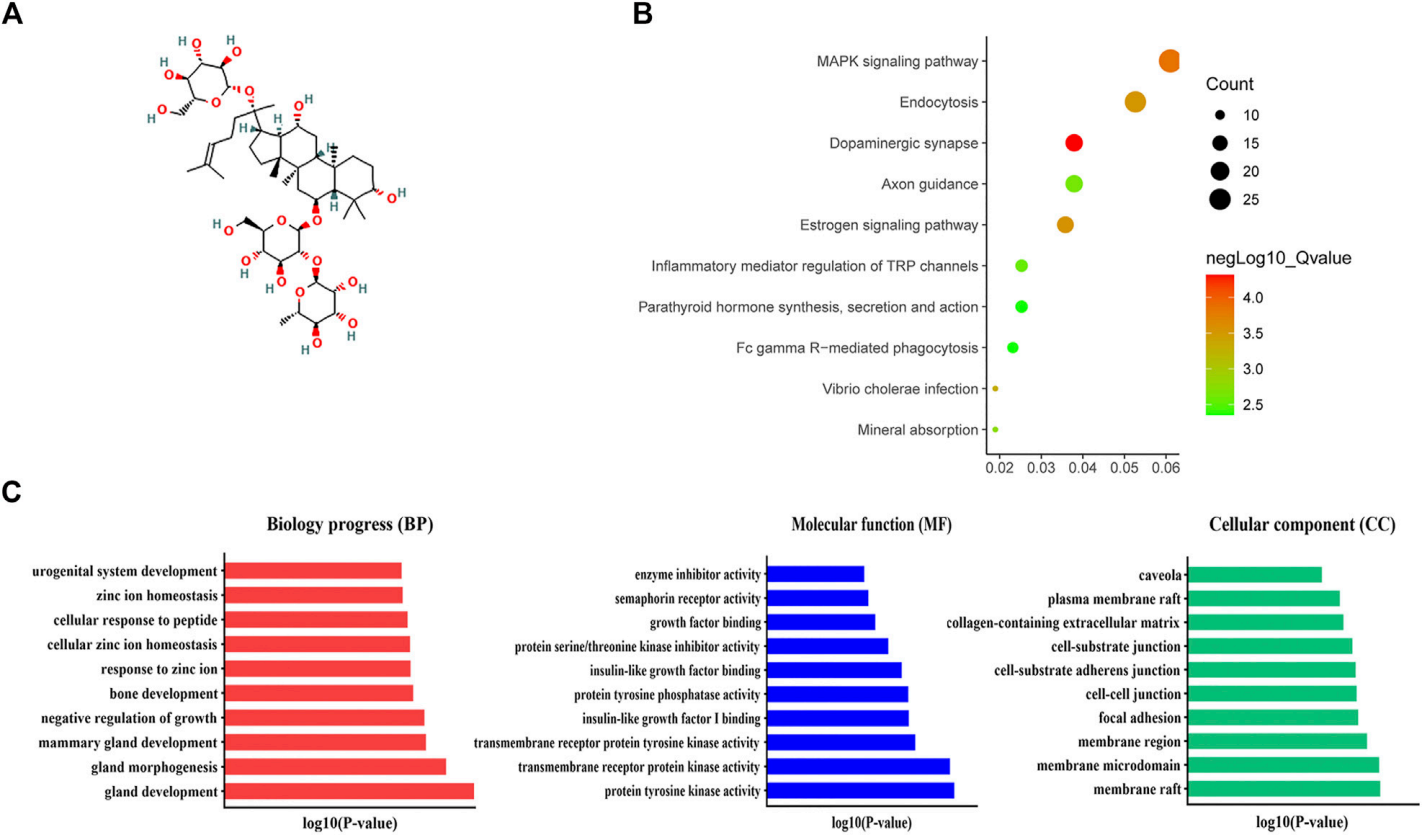
KEGG and Go analysis. **(A)** Structural formula of ginsenoside Re. **(B)** Revealed the 10 significantly enriched pathways in KEGG analysis. **(C)** The top terms of molecular functions (MF), biological processes (BP), and cellular components (CC) in GO enrichment analysis.

Functional Enrichment Analysis of Ginsenoside Re, the Main Ingredient of Guilingji The structural formula of ginsenoside Re is shown in Figure 5A. Evaluation of drug efficacy and discovery of new drug targets are the characteristics of pharmacotranscriptomics (Fang et al., 2021). Therefore, it is necessary not only to identify the active ingredients, but also to further explore their therapeutic targets. Hence, GO analysis and KEGG were performed to assess key molecules and pathways. As shown in Figure 5B, MAPK signaling pathway, endocytosis and dopaminergic synapse were mainly enriched. GO analysis revealed that gland development, gland morphogenesis, mammary gland development, and negative regulation of growth were the primary terms involved in BP; membrane microdomain, membrane raft, and membrane region were significantly enriched in CC; protein tyrosine kinase activity, transmembrane receptor protein tyrosine kinase activity, transmembrane receptor protein kinase activity, and insulin-like growth factor I binding were top enriched in MF (Figure 5C).

### Guilingji Inhibits the Phosphorylation of MAPK Pathway Proteins in the Testes of Immp2l Mutant Mice

**FIGURE 6:**
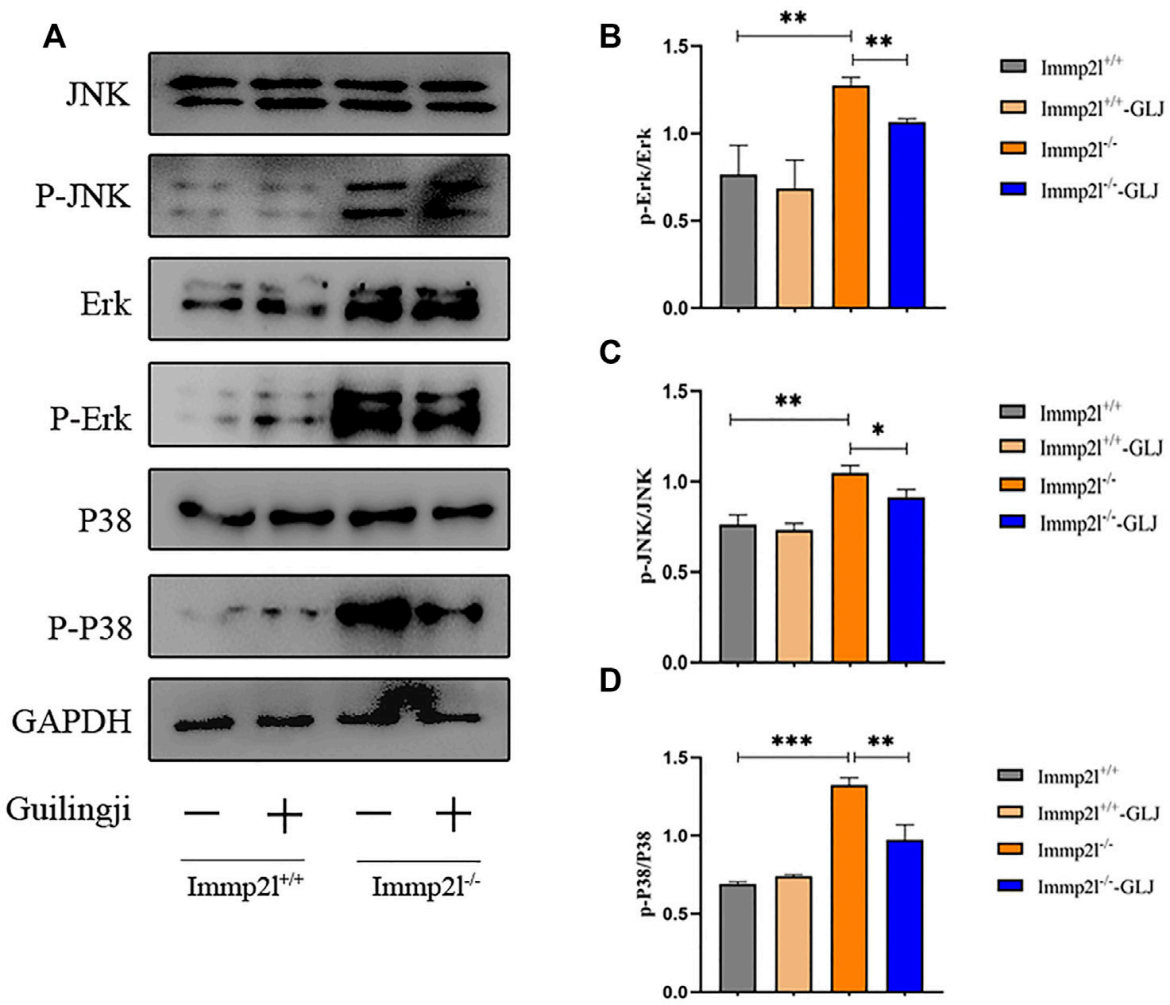
The effects of Guilingji on the expression of proteins related to MAPK signaling pathways. **(A)** Relative expression of p-JNK, p-p38 and p-ERK was assayed through western blot. **(B**–**D)** Quantitation of relative protein levels of p-JNK, p-P38 and p-ERK respectively. The bars showed means ± SEM of three independent experiments. *p < 0.05, **p < 0.01, ***p < 0.001. *n* = 3.

Previous studies have shown that activation of MAPK pathways is closely related to oxidative stress-related apoptosis (Wang et al., 2019). Pathway enrichment analysis suggested that MAPK signaling pathway may play a role in the administration of Guilingji. We hypothesized that Guilingji might play an antioxidative role through the inhibition of the MAPK signaling pathways. In this study, the expressions of p38, p-p38, ERK, p-ERK, JNK, and p-JNK in the MAPK subfamily were detected by western blotting. As shown in Figure 6, the expression level of phosphorylated proteins in the MAPK pathways was remarkably increased in Immp2l^-/-^ mice. However, compared with the untreated group, the levels of phosphorylated proteins in MAPK pathway were decreased in the treatment group (*p* < 0.05). In conclusion, these results suggested that Guilingji may play a protective role in spermatogenesis by inhibiting the above signal pathway.

## Discussion

In this study, we showed that the level of oxidative stress in Immp2l mutant mice testis was higher compared with wild-type mice, leading to spermatogenic impairment. We revealed that the Guilingji administration reduced ROS level, inhibited germ cell apoptosis and improved spermatogenic function. Importantly, we found that the therapeutic effects of Guilingji was depended on MAPK pathways. In summary, we elaborated the therapeutic effects of Guilingji administration in oligospermia animal models, which was depended on the ant-oxidative capacities.

As we know, oxidative stress refers to the imbalance between the oxidative system and the antioxidant system, which is the main factor in the pathology of infertility in 30–80% of infertile men (Aitken and Fisher, 1994; Agarwal et al., 2006; Barati et al., 2020). Compared with fertile men, infertile men show higher levels of semen oxidative stress and increased apoptosis. Poor sperm condensed chromatin (due to elevated ROS levels and high levels of oxidative stress) entering the apoptotic cascade, a lot of apoptotic germ cells in testis or Phosphatidylserine externalized cell, high levels of caspase activation and DNA damage, it is considered to be the main causes of male infertility (Bisht et al., 2017). In our study, Immp2l mutant mice exhibit the same phenotype, with high oxidative stress level, increased apoptosis level and spermatogenic dysfunction in testis. Some studies have already demonstrated that mitochondrial superoxide level was increased in Immp2l mutant mice, which lead to spermatogenic damage and infertility (George et al., 2012). These evidences suggest that oxidative stress is an important mediator of infertility and Immp2l^-/-^ mice act as one of the ideal animal models of infertility, alleviating oxidative stress is conducive to ameliorating spermatogenesis of Immp2l mutant mice.

Previous studies have shown that red ginseng and ginsenosides can decrease and/or eliminate the production of free radicals by modulating the activity of antioxidase (Shi et al., 2013). While red ginseng and ginsenosides are the main herbs contained in Guilingji. And the total amount of ginsenoside Re (C48H82O18) is more than 100 ug in Guilingji capsule (Zhao et al., 2020). The multiple pharmacological effects of Guilingji have been confirmed by other study groups, including antioxidation, anti-inflammation, anti-aging and so on (Zhao et al., 2019; Du et al., 2020; Zhao et al., 2020). Accordingly, we speculate that Guilingji can protect the testis from apoptosis under oxidative stress.

Intracellular antioxidants such as glutathione (GSH), superoxide dismutase (SOD) protect cells against oxidative damage by eliminating cytoplasmic ROS (Nakamura et al., 2010). Guilingji obviously increased the activities of above enzymes, which may reduce the accumulation of ROS and subsequently reduce the oxidative damage to biological macromolecules. Malondialdehyde (MDA) is an important lipid peroxidation product, which can be used as an index to detect the oxidative damage of cell membrane. And it is a measure of peroxidation damage to spermatozoa under the condition of oxidative stress (Aitken et al., 2013; Dong et al., 2015). We found a decrease of MDA levels in the Guilingji-treatment compared with that control group, indicating the reduced function on lipid peroxidation that occurred in the Immlp2l mutant mice.

The apoptotic cascade is caused by excessive production of ROS and oxidative stress, which hinder the basic functions of sperm, thereby making sperm inactive and immotile. Therefore, the development of therapeutic methods to improve or inhibit sperm cell apoptosis induced by oxidative stress is an urgent requirement (Greco et al., 2005). In this study, there is evidence that when oxidative stress is inhibited, apoptosis can be significantly reduced. The result of western blot shown that the expression of pro-apoptotic proteins, including cleaved caspase-3, and Bax, were increased. while the anti-apoptotic protein Bcl-2 was down-regulated. We believe that Guilingji can protect against mitochondrial dysfunction by regulating oxidative stress, thereby reducing the activation of caspase and inhibiting cell apoptosis.

The separation of active ingredients from TMC and further analysis of their mechanism of action are of great significance for discovering drug targets and evaluating drug efficacy (Fang et al., 2021). Therefore, we downloaded data from HERB database, and the pathway enrichment analysis found that MAPK signaling pathway was mainly enriched. Furthermore, there is also the mechanism of apoptosis may be related to the activation of p38/MAPK and JNK/MAPK signal transduction pathways (Ashwell, 2006; Liu et al., 2016). MAPK is composed of three main subgroups: extracellular signal-regulated kinase (ERK), p38-MAPK, and c-Jun N-terminal kinase (JNK) (Yue and López, 2020). Accumulating evidence has identified MAPK pathways are activated by oxidative stress in cells and tissues. Importantly, MAPK signaling pathways are involved in regulating reproductive functions in the testis (Sinha Hikim et al., 2003; Johnson et al., 2008; Li et al., 2009). For example, Liu B et al. found that PM2.5 disrupted spermatogenesis via ROS-mediated MAPK pathways, while antioxidants (vitamin C and E) can inhibit the MAPK pathways and improve testicular spermatogenesis (Liu et al., 2019). Li M et al. mentioned that it can alleviate the damage of BTB and spermatogenesis caused by cadmium through inhibiting the unnecessary activation of MAPK pathways in Sertoli cells (Li et al., 2009). What’s more, some studies revealed that administration of antioxidants can alleviate oxidative stress and injury via MAPK pathways (Li et al., 2020; Ye et al., 2020). In our research, Guilingji treatment reduced the high phosphorylation levels of p-38, JNK and ERK1/2 in Immp2l^-/-^ mice, demonstrate that Guilingji strongly inhibit the MAPK signal transduction pathways. All in all, our experiment revealed that Guilingji could attenuated oxidative stress and apoptosis in Immp2l mutant mice via restrain activation of the MAPK signaling pathways.

## Conclusion

In short, the results of this study systematically proved that Guilingji can attenuate ROS, oxidative stress and apoptosis by inhibiting MAPK-mediated signal pathways in the immp2l mutant mouse model. This study provides evidence that Guilingji may be effective in the clinical treatment of male infertility. However, the use of a single dose of Guilingji is deficiency of our experiment, which will be improved in future studies. In addition, the regulation mechanism of Guilingji on metabolites needs further experimental verification.

## Data Availability

The original contributions presented in the study are included in the article/Supplementary Material, further inquiries can be directed to the corresponding authors.
